# Polymer Informatics at Scale with Multitask Graph Neural Networks

**DOI:** 10.1021/acs.chemmater.2c02991

**Published:** 2023-02-15

**Authors:** Rishi Gurnani, Christopher Kuenneth, Aubrey Toland, Rampi Ramprasad

**Affiliations:** School of Materials Science and Engineering, Georgia Institute of Technology, 30332 Atlanta, Georgia United States

## Abstract

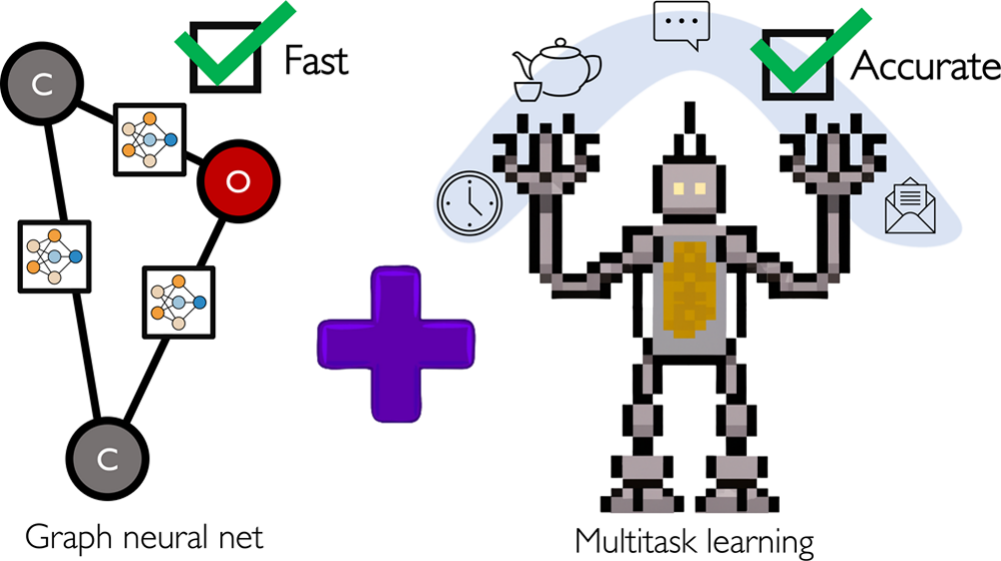

Artificial intelligence-based methods are becoming increasingly effective at screening libraries of polymers down to a selection that is manageable for experimental inquiry. The vast majority of presently adopted approaches for polymer screening rely on handcrafted chemostructural features extracted from polymer repeat units—a burdensome task as polymer libraries, which approximate the polymer chemical search space, progressively grow over time. Here, we demonstrate that directly “machine learning” important features from a polymer repeat unit is a cheap and viable alternative to extracting expensive features by hand. Our approach—based on graph neural networks, multitask learning, and other advanced deep learning techniques—speeds up feature extraction by 1–2 orders of magnitude relative to presently adopted handcrafted methods without compromising model accuracy for a variety of polymer property prediction tasks. We anticipate that our approach, which unlocks the screening of truly massive polymer libraries at scale, will enable more sophisticated and large scale screening technologies in the field of polymer informatics.

## 1 Introduction

Polymers have emerged as a powerful class of materials for a wide range of applications because of their low-cost processing, chemical stability, tunable chemistries, and low densities. These attributes have led to vigorous, widespread, and sustained research, and to the development of new polymeric materials.^1−3^ The result is a constant flux of materials data. Over the past decade, the polymer informatics community has translated this data stream into machine-learned property predictors that efficiently screen libraries of candidate polymers for subsequent experimental inquiry.^4,5^

Currently, most approaches for polymer screening rely on handcrafted features—extracted from the chemical structure of a polymer repeat unit—as input for property predictors.^6,7^ These approaches are highly accurate, but feature extraction becomes a bottleneck (as discussed in Section 3.1) when used to screen vast swathes of the polymer chemical space. This bottleneck is increasingly exposed by the proliferation of enumeration methods^8,9^ and long-sought^10,11^ inverse predictors,^12−16^ which directly locate optimal pockets of the chemical space from a user-defined wish list of material properties. By leveraging these tools, the day that we routinely generate billions of polymer candidates is fast approaching. Advances in polymer screening and feature engineering are needed to keep up with this pace.

An alternative to handcrafting features is to “machine learn” them. One approach is to represent the material as raw text, such as a simplified molecular-input line-entry system (SMILES)^17^ or BigSMILES^18^ string, and then learn features with a neural network specifically designed for natural language processing.^19^ Another promising approach is to represent the material as a graph, and then train a Graph Neural Network (GNN)^20^ to learn features. To date, GNNs have outperformed approaches based on handcrafted features^20−24^ on the massive QM9 database^25^ for small molecules. Similarly, feature learning approaches have supplanted traditional methods in other domains (e.g., convolutional neural networks^26^ in computer vision and transformers^27^ in natural language processing) where the extraction of handcrafted representations from the input data is nontrivial or impractical.^26^

Another important emerging trend in machine learning (ML) for materials science is multitask learning.^5,28^ The central concept of multitask learning is that by training a model to learn multiple correlated target properties at the same time, the risk of overfitting to any one target property is reduced, leading to improved predictive performance for each property.^28^ A similar effect can also be observed in nature. For example, there is evidence that training in one sport can improve a young athlete in another related sport.^29^

**Figure 1:**
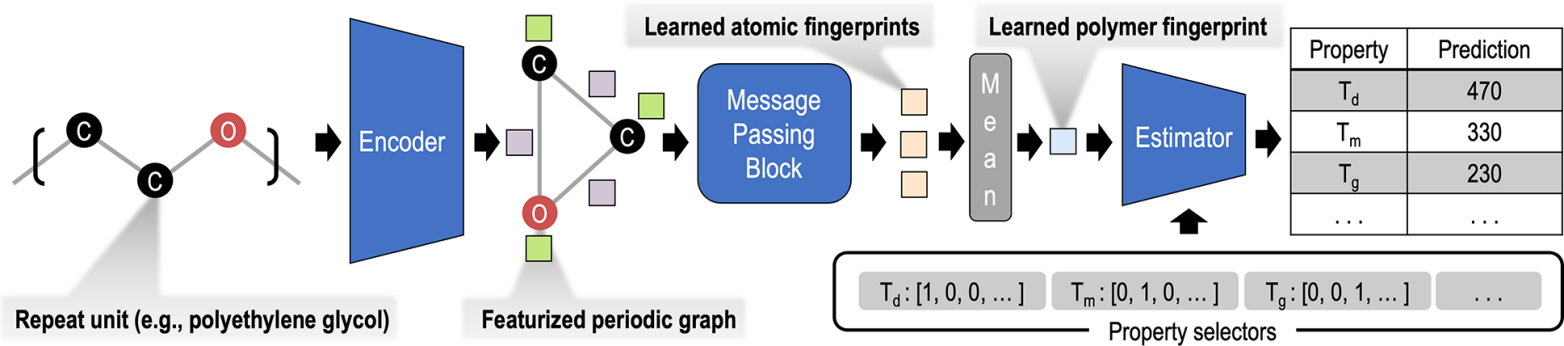
PolyGNN architecture. The Encoder converts the repeat unit SMILES string to a periodic graph and then computes initial atomic and bond fingerprint vectors (green and purple squares, respectively). A subsequent set of atomic fingerprints (yellow squares) are learned by the Message Passing Block and then averaged, yielding the learned polymer fingerprint (light blue square). This fingerprint and a series of selector vectors are passed to the Estimator, producing a series of property predictions. *T*_d_, *T*_m_, *T*_g_ refer to the critical temperatures for thermal decomposition, melting, and glass transition, respectively.

A handful of polymer GNNs have been explored in the past.^30−36^ The majority of these approaches are single task. The GNN proposed by Mohapatra et al.^34^ is suitable for biopolymers, in which the monomer sequence is known. Other approaches,^32,33,35,36^ geared toward synthetic polymers (the subject of interest in this work), represent a polymer using the graph of a predominant repeat unit. This introduces the need for invariance to certain transformations of the repeat unit graph: translation, addition, and subtraction (as defined in Section 2.2). A subset of the GNNs for synthetic polymers^35,36^ are invariant to translation, but not to addition and subtraction. In other words, a GNN that preserves the invariant properties of polymer repeat units has not been developed until now. Our work, a powerful multitask GNN architecture (see Figure 1) for polymers, fills this gap. We call this development the Polymer Graph Neural Network (polyGNN).

In the small molecule domain, the adoption of GNNs is motivated by systematic work^20^ comparing GNNs and handcrafted approaches on even footing across a diverse set of molecules and predictive tasks. Analogous studies are absent from the synthetic polymer domain. Previous works have compared feature learning and handcrafted approaches for up to two^31,35^ polymer properties, or for several properties in the same class^30^ (e.g., electronic properties). In what follows, we compare polyGNN with the handcrafted fingerprint originally hosted under the Polymer Genome (PG) project^4^ on a large and diverse data set consisting of more than 13,000 polymers and 30+ predictive tasks—spanning thermal, thermodynamic, physical, electronic, optical, dielectric, and mechanical properties, the Hildebrand solubility parameter, as well as permeability of six gases.

Our benchmark, the PG fingerprint, contains descriptors that correspond to one of three length scales. The finest-level components are atomic triples (e.g., C_*i*_O_*j*_N_*k*_) where the subscripts denote the atomic coordination. The next (block) level contains predefined atomic fragments (e.g., the common cycloalkenes). These two levels contain strictly one-hot features. At the highest (chain) level are numerical features that describe the atomic or block topology (e.g., the number of atoms in the largest side chain). The handcrafted PG fingerprint is the current state-of-the-art in polymer representation and has shown success in the numerical representation of materials over a wide chemical and property space.^4,8,37^ The handcrafted PG fingerprint-based property predictors thus serve as veritable performance baselines. We find that polyGNN, relative to these baselines, leads to a 1–2 orders of magnitude faster fingerprinting and better or comparable model accuracy in most polymer property prediction tasks. polyGNN thus offers a powerful new polymer informatics option for screening the polymer chemical space at scale.

## 2 Methods

### 2.1 Data Set and Preparation

**Figure 2:**
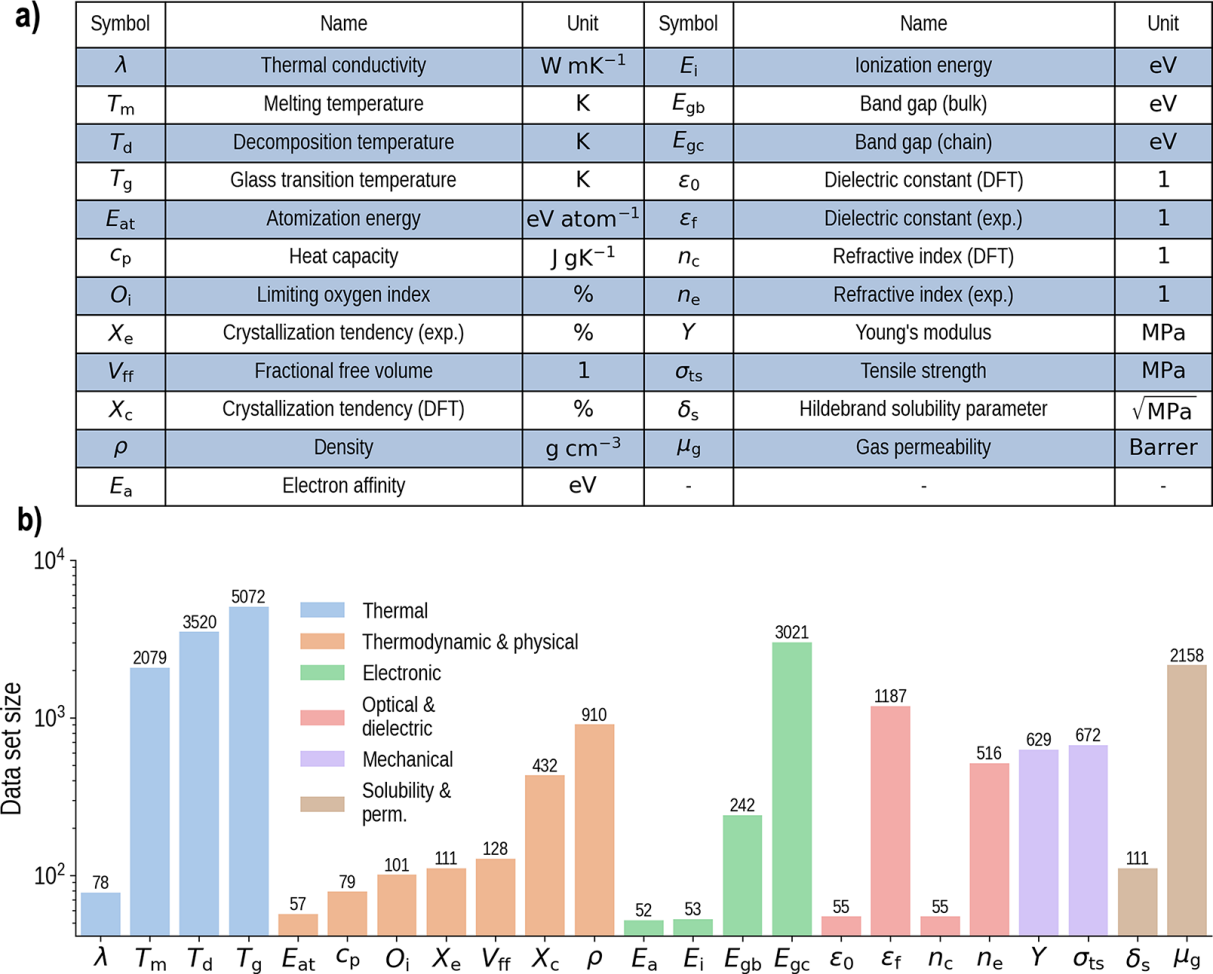
Breakdown of our data set. (a) The symbol, name, and unit of each property in our data set. For properties with data from both experiment and DFT calculations, the two sources are distinguished by the abbreviations “expt.” and “DFT”. Our data set includes the permeability μ_*g*_ of six gases *g* ∈ {He, H_2_, CO_2_, O_2_, N_2_, CH_4_}. Each permeability data point is scaled by *x* → log_10_(*x* + 1). Our experimental dielectric constant ϵ_*f*_ data contains measurements at nine frequencies *f* ∈ {1.78, 2, 3, 4, 5, 6, 7, 9, 15} in log_10_ Hz. The distributions of μ_*g*_ and ϵ*_f_* are given in Section S1. (b) The data set size per property, shown on both the *y*-axis and above each bar. Bars of the same color belong to the same property class. “perm.” stands for gas permeability.

Our corpus contains measurements for up to 36 properties of 13,388 polymers, yielding over 21,000 data points in total. The unit and symbol for each property is listed in Figure 2a. The distribution of data points per property is plotted in Figure 2b. These data points come from in-house density functional theory (DFT) computations,^38−40^ experimental data collected from the literature,^41−46^ printed handbooks,^47−49^ and online databases.^50,51^ Band gaps were calculated for both individual polymer chains *E*_gc_ and polymer crystal (bulk) structures *E*_gb_ using DFT. DFT data contain uncertainties due to the choice of exchange correlation functional, pseudopotentials, optimization procedure, etc., while data from physical experimentation comes with uncertainty due to sample and measurement conditions. Thus, data for the same property but from different sources (e.g., DFT-computed and experimentally measured refractive index) are separated and then colearned with multitask learning.

Our multitask learning approach requires data preprocessing steps. First, the training data for each property was MinMax scaled between zero and one. This ensures that the optimizer of a multitask ML model equally weights the loss for each property during training. Second, to better exploit correlations between properties,^5^ we divided our entire 36 property data set into six “property groups”: thermal properties, thermodynamic and physical properties, electronic properties, optical and dielectric properties, solubility and gas permeability, and mechanical properties. The stratification of properties by group is shown in Figure 2b. Finally, we designate each property within one group a unique one-hot “selector” vector (see Figure 1 for example selector vectors of thermal properties). These vectors are used by our ML models to distinguish between multiple tasks.

### 2.2 polyGNN

All GNNs rely on a well-defined graph representation of their input. If the input is a small molecule, then building a corresponding graph is straightforward—each heavy (i.e., non-hydrogen) atom is a graph node and each bond between heavy atoms is a graph edge. However, polymers are macromolecules with numerous atoms and bonds. Creating a node and edge for each atom or bond will generate a massive graph. Machine learning based on thousands of such graphs would be computationally inefficient. Instead, we construct a polymer graph from its repeat unit alone and propose that additional information (e.g., molecular weight, end groups, etc.)—if available—be concatenated to each computed atom or bond fingerprint and/or to the learned polymer fingerprint.

Ideally, our learned polymer fingerprint must respect the invariances present in a polymer repeat unit. We identify three key transformations—translation, addition, and subtraction—that repeat units of infinite 2D polymer chains are invariant to. We define translation as the movement of the periodicity window, which can produce periodic repeat units that are all equivalent. For example, (−OCC−), (−COC−), and (−CCO−) are equivalent repeat units of polyethylene glycol, related to one another by translation. We define addition (subtraction) as the extension (reduction) of a repeat unit by one or more minimal repeat units. For example, (−COCO−) and (−COCOCO−) are equivalent repeat units, related to one another by the addition (or subtraction) of their minimal repeat unit, (−CO−). We have constructed polyGNN to be invariant under such transformations, as discussed below.

The polyGNN architecture is composed of three main modules: an Encoder for processing the repeat unit, a Message Passing Block for fingerprinting, and an Estimator to colearn multiple properties. In the polyGNN Encoder, bonds are added between heavy atoms at the boundary of any input repeat unit, forming a periodic polymer graph (as shown in Figure 1). This ensures that the graph of the repeat unit, and hence its learned fingerprint, is invariant to translation. Then, each atom and bond in the graph are given initial feature vectors (described later in Section 2.3) that are computed using RDKit.^52^ The featurized graph is passed to the Message Passing Block and then to the aggregation function. In the Message Passing Block, the initial feature vectors are passed between neighboring atoms. This information flow is the mechanism by which rich polymer features are learned (described later in Section 2.4).

**Figure 3:**
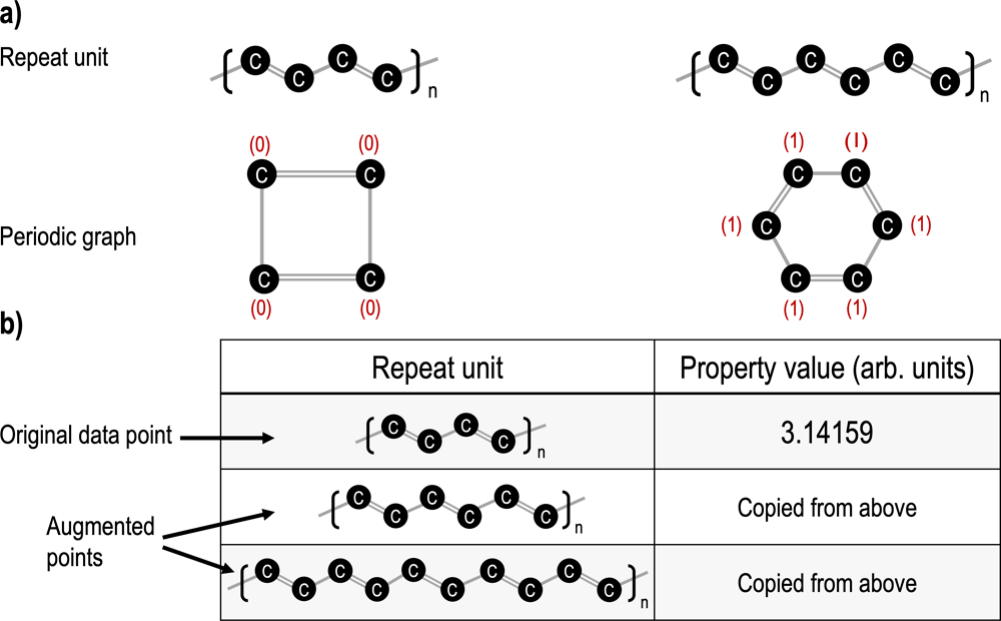
Overview of data set augmentation. (a) Two equivalent repeat units of infinite polyacetylene and their corresponding periodic graphs. Each atom in the graph is labeled with a zero if the atom is aliphatic or labeled with a one if the atom is aromatic. Other atomic features and all bond features are not shown for visual clarity. (b) Data augmentation strategy for polyGNN. Rows of the original training data are transformed by repeat unit addition.

After message passing, the sequence of learned atomic fingerprints is aggregated into a single polymer fingerprint by taking the mean. Taking the mean rather than the sum ensures that, for example, (−COCO−) and (−COCOCO−) are mapped to the same fingerprint. However, there are polymers (see Figure 3a) where the desired invariance is not preserved. These conflicts arise because RDKit treats periodic polymer graphs as cyclic molecules. To address these conflicts, we propose two approaches. In the first approach, which we will continue to refer to as polyGNN, the original training data set is augmented with transformed repeat units (see Figure 3b). Thus, although polyGNN is not invariant to addition or subtraction in these complicated cases, it achieves approximate invariance after learning from augmented data. This choice was inspired by state-of-the art image classification models, which are trained using cropped and flipped images.^53^ In this work, we find that data augmentation is also effective for training polyGNNs but does increase training time—a one-time cost. As an alternative, we created a variant, polyGNN2, with guaranteed invariance to addition and subtraction (and thus no need for augmentation). Invariance is achieved by modifying the Encoder to compute features on an extended polymer graph instead of on the periodic graph (see Section S2). However, operating on the extended graph notably slows fingerprinting in polyGNN2, and so we instead focus on polyGNN in what follows.

### 2.3 Fingerprinting Graphs

The node features used in this work are element type, node degree, implicit valence, formal charge, number of radical electrons, hybridization, aromaticity (i.e., whether or not a given node is part of an aromatic ring), and number of hydrogen atoms. The edge features are bond type, conjugation (i.e., whether or not a given edge is part of a conjugated system), and ring member (i.e., whether or not a given edge is part of a ring).

### 2.4 Neural Message Passing

In GNNs, “messages” between neighboring atoms in a graph are iteratively passed along chemical bonds. After each iteration, every atom fingerprint is updated using the messages. In this way, atoms learn about their local neighborhood over time. By fitting parameters (e.g., weights and biases) in the model, the information contained in each message is optimized for the task at hand. This process is captured by three general but abstract equations presented in Section S3. In this section, for concreteness, we will demonstrate message passing using a highly simplified example.

First, consider the graph of infinite polyethylene glycol (PEG), shown in Figure 1. We restrict our initial atom features to the element type and our initial bond features to the bond type. Thus, all edge fingerprints on the PEG graph are set to [1, 0, 0, 0] (indicating the presence of single bonds and no double, triple, or aromatic bonds). The two carbon atoms in PEG are initialized with a fingerprint of [1, 0] (indicating the presence of C atoms and not O atoms). We index these two nodes 0 and 1. The oxygen atom, with index 2, in PEG is initialized with a fingerprint of [0, 1]. Now, we compute messages **m**_*i*,*j*_ between all pairs of chemically bonded atoms using the functional form
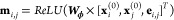
where *i*, *j* are atom indices, **x**_*i*_^(0)^ is an initial atom fingerprint, and **e**_*i,j*_ is a bond fingerprint. Note that, for simplicity, we ignore bias terms and use the Rectified Linear Unit (ReLU) activation in this example. **W_ϕ_** is a matrix of parameters. Before training, the parameters are randomly initialized. During training, the parameters are iteratively updated (i.e., learned) using some flavor of stochastic gradient descent. In this example, our choice of initial parameters will be guided by mathematical convenience, and we do not consider subsequent weight updates. Choosing
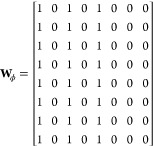
gives us
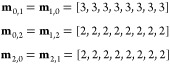


Now, these messages can be used to update the fingerprint of each atom using the functional form
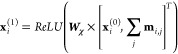
where ***W*_χ_** is a matrix of parameters, and *j* takes on values corresponding to atoms that share a chemical bond with atom *i*. After we conveniently initialize *W*_χ_ to a 2 × 10 all-ones matrix, we have
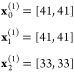


So, by exchanging messages with neighbors, the fingerprint of each carbon atom in PEG was updated from [1, 0] to [41, 41] and the fingerprint of each oxygen atom was updated from [0, 1] to [33, 33]. The effect of message passing is clear. Initially, the oxygen atom was not aware of neighboring carbon atoms (that is, **x**_2,2_^(0)^ = 0, where **x**_*i,l*_ is the *l*^th^ dimension of **x**_*i*_). However, after passing one round of messages, the oxygen atom becomes aware of its carbonaceous neighbors (i.e., **x**_2,2_^(1)^ ≠ 0). Likewise, the carbon atoms become aware of their neighboring oxygen atom over time.

## 3 Results and Discussion

### 3.1 Benchmarking Speed

polyGNN was developed with a primary objective in mind: to increase the rate at which large libraries of polymers may be screened. We quantified this rate by measuring the time needed to fingerprint a data set of 13,338 known polymers on a variety of different capacities and hardware. Capacity, as used in this work, is a hyperparameter that specifies both the number of message passing steps and the depth of each multilayer perceptron (MLP) in the network.

**Figure 4:**
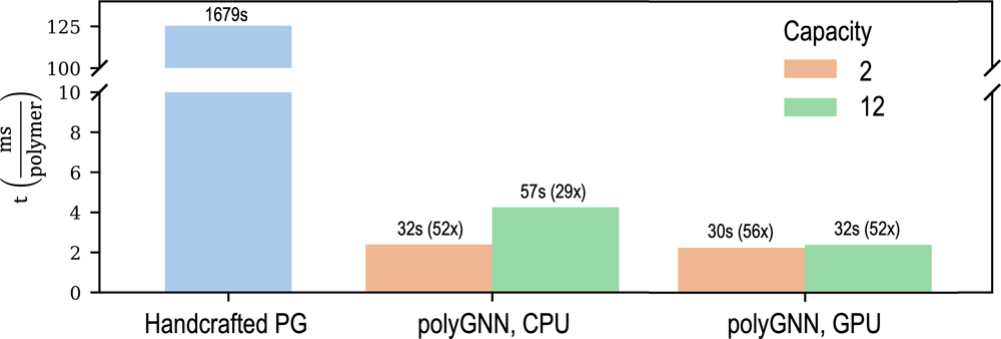
Fingerprint time as a function of method, capacity, and hardware. Fingerprint time *t*, measured in milliseconds per polymer, is plotted on the *y*-axis. *t* was computed using a diverse set of 13,388 polymers. Above each bar is the total time (in plain text) in seconds taken to compute fingerprints for the entire set as well as the speed up (in parentheses) relative to the handcrafted PG method. Method and hardware are labeled on the *x*-axis. CPU and GPU refer to one Intel Xeon Gold 6140 CPU core and to one 32 GB Nvidia Tesla V100-PCIE GPU, respectively. Capacity is denoted by bar color.

Figure 4 presents the computation times for generating 13,388 polymer fingerprints using a randomly initialized polyGNN model. A shallow polyGNN (with a capacity of two) fingerprints the set of polymers in 32 s (2.4 ms per polymer) on one CPU or 30 s (2.2 ms per polymer) on one GPU. Meanwhile, a deep polyGNN (with a capacity of 12) takes 57 s (4.3 ms per polymer) to compute the fingerprint set on one CPU or 32 s on one GPU. For each of the above, the time spent on the Encoder was fixed at 26 s. The remaining time was spent on the Message Passing Block which, unlike the Encoder, can run on either CPUs or graphics processing units (GPUs).

By extrapolation, this means that fingerprinting a library of 1 billion polymers using polyGNN would take 26 days in the best case (shallow model run on a GPU) and 47 days in the worst case (deep model run on one CPU). Meanwhile, at a rate of 125.4 ms per polymer, fingerprinting a library of 1 billion polymers would take nearly 4 years on one CPU using the handcrafted PG approach. Of course, the rates for either approach can be further sped up with parallelization and/or increased random access memory.

### 3.2 Benchmarking Accuracy

Here we evaluate the predictive accuracy of polyGNN models on 34 of the 36 properties in our data set; dielectric constants at 10^7^ and 10^9^ Hz (ϵ_7_ and ϵ_9_) were excluded because our corpus contains fewer than 50 data points for these properties. The data for the remaining properties was divided into a training and a test set in a 4:1 ratio, with three such random divisions carried out per property for the purpose of computing statistics of model performance, such as the mean and standard deviation of the root-mean squared-error (RMSE).

Kuenneth et al.^5^ showed that multitask learning significantly improves the accuracy of polymer property prediction, relative to single task learning. Thus, we train single task (ST) and multitask (MT) polyGNNs and compare both on the same data. As a benchmark, we also train both ST and MT “PG-MLPs” (i.e., MLPs that use the handcrafted PG fingerprint as input; see Section S4 for details on this architecture). A detailed discussion of our training procedure can be found in Section S5. The RMSE and *R*^2^ values of polyGNN and PG-MLP are compared in Tables 1 and S1.

**Table 1:** Average RMSE Plus/Minus One Standard Deviation on Unseen Test Data^a^

Property	MT polyGNN	MT PG-MLP	ST polyGNN	ST PG-MLP
λ*	**0.0547** ± 0.0103	0.0630 ± 0.0082	0.0580 ± 0.0096	0.0663 ± 0.0201
*T*_m_	**45.0** ± 1.8	**47.2** ± 2.2	55.3 ± 2.8	53.1 ± 1.3
*T*_d_	**58.7** ± 3.3	**59.3** ± 2.0	67.7 ± 3.2	71.9 ± 6.9
*T*_g_	**31.7** ± 1.5	**34.0** ± 0.9	**36.6** ± 1.0	**35.5** ± 1.6
				
*E*_at_*	**0.114** ± 0.071	0.284 ± 0.089	**0.0913** ± 0.0224	0.155 ± 0.040
*c*_p_*	**0.172** ± 0.033	0.223 ± 0.085	**0.171** ± 0.019	**0.161** ± 0.030
*O*_i_*	**8.99** ± 1.01	9.77 ± 1.57	**8.79** ± 0.46	**8.63** ± 0.47
*X*_e_*	15.0 ± 3.7	**13.1** ± 4.6	15.8 ± 3.9	17.1 ± 5.1
*V*_ff_*	0.0380 ± 0.0191	0.0423 ± 0.0216	**0.0330** ± 0.0182	0.0373 ± 0.0215
*X*_c_	**16.6** ± 1.3	**17.4** ± 2.5	18.6 ± 1.9	19.1 ± 2.2
ρ	**0.0640** ± 0.0053	0.0937 ± 0.0025	**0.0627** ± 0.0015	0.385 ± 0.264
				
*E*_a_*	0.380 ± 0.034	0.483 ± 0.148	**0.341** ± 0.055	**0.357** ± 0.107
*E*_i_*	**0.540** ± 0.170	0.678 ± 0.231	59.9 ± 102.5	0.676 ± 0.139
*E*_gb_*	**0.468** ± 0.066	**0.535** ± 0.123	0.716 ± 0.164	0.737 ± 0.058
*E*_gc_	**0.445** ± 0.018	**0.491** ± 0.033	**0.442** ± 0.020	**0.494** ± 0.026
				
ϵ_0_*	**0.285** ± 0.101	**0.284** ± 0.061	0.362 ± 0.086	**0.252** ± 0.014
ϵ_1.78_*	0.427 ± 0.042	**0.328** ± 0.067	1.34 ± 0.30	0.988 ± 0.517
ϵ_2_*	0.478 ± 0.228	**0.376** ± 0.257	2.67 ± 2.78	0.937 ± 0.201
ϵ_3_*	**0.621** ± 0.250	0.806 ± 0.338	1.39 ± 0.21	1.42 ± 0.22
ϵ_4_*	**0.284** ± 0.018	**0.252** ± 0.030	0.650 ± 0.108	0.602 ± 0.175
ϵ_5_*	**0.212** ± 0.023	**0.243** ± 0.011	0.479 ± 0.266	0.658 ± 0.358
ϵ_6_*	0.323 ± 0.075	**0.274** ± 0.034	0.676 ± 0.315	0.487 ± 0.214
ϵ_15_	**0.125** ± 0.015	0.145 ± 0.019	0.144 ± 0.021	0.171 ± 0.027
*n*_c_*	**0.0507** ± 0.0186	0.0733 ± 0.0191	0.0933 ± 0.0304	0.0957 ± 0.0251
*n*_e_	**0.0413** ± 0.0023	**0.0437** ± 0.0090	0.0540 ± 0.0087	0.0760 ± 0.0262
				
*Y*	**0.827** ± 0.099	**0.760** ± 0.169	0.877 ± 0.074	0.860 ± 0.196
σ_ts_	**23.3** ± 5.5	**22.2** ± 3.9	28.1 ± 4.6	25.8 ± 3.9
				
δ_s_*	**1.15** ± 0.11	2.11 ± 0.10	1.65 ± 0.33	1.36 ± 0.09
μ_He_*	**0.133** ± 0.017	**0.111** ± 0.014	0.265 ± 0.065	0.246 ± 0.011
μ_H_2__*	**0.127** ± 0.006	**0.104** ± 0.011	0.287 ± 0.013	0.367 ± 0.034
μ_CO_2__	**0.166** ± 0.015	**0.161** ± 0.019	0.430 ± 0.025	0.525 ± 0.212
μ_CH_4__	**0.132** ± 0.024	**0.113** ± 0.023	0.366 ± 0.030	0.397 ± 0.006
μ_N_2__	**0.124** ± 0.011	**0.109** ± 0.018	0.410 ± 0.104	0.397 ± 0.038
μ_O_2__	**0.139** ± 0.014	**0.114** ± 0.004	0.399 ± 0.062	1.83 ± 2.46

a Properties marked with an asterisk contain 300 or fewer data points. Models with the best, or comparable with the best, average RMSE are bolded. The unit of each RMSE value matches those listed in Figure 2a; for example, the RMSE of the MT polyGNN approach on *T*_g_ is 31.7 ± 1.5 K.

We note several observations from these results. First, our data augmentation strategy plays a critical role in teaching polyGNN models invariance to addition and subtraction (see Table S2). Second, we find that MT learning is an important component of our approach, especially in low data situations. As shown in Table S1, polyGNNs that do not use MT learning exhibit erroneous predictions (i.e., negative *R*^2^ value) for five properties—*E*_i_, ϵ_1.78_, ϵ_2_, ϵ_5_, ϵ_6_—each with 158 or fewer data points. In contrast, with MT learning, polyGNNs exhibit positive *R*^2^ for each of the 34 properties studied.

Third, we find that polyGNNs tend to exbihit better or comparable accuracy than PG-MLPs, especially when the number of training data points is greater than 300. For the 14 properties containing more than 300 data points, each MT polyGNN model is either more accurate than or comparably accurate to its corresponding MT PG-MLP model (we define two models as having comparable accuracy for a property if the difference in average RMSE of their predictions is within 5% of that property’s standard deviation σ, see Table S3 for a complete list of σ values). However, for the 20 properties containing 300 data points or less, the situation becomes more complex. MT polyGNN models still perform well relative to the MT PG-MLP benchmark, but not for every property. MT polyGNN models are more or comparably accurate for 16 properties but are notably less accurate on four properties (experimental crystallization tendency *X*_e_, ϵ_1.78_, ϵ_2_, ϵ_6_).

The relatively low performance on these four properties could be explained by the fact that the polyGNN models trained here struggle to learn the block- or chain-level features (which typically consist of 4+ atoms) present in the handcrafted PG fingerprint. In principle, increasing the number of message passing steps—so as to capture larger length scale features—should mitigate this challenge. In practice, however, we observe a threshold number of message passing steps. Above three message passing steps, model generalization only worsens—regardless of the property of interest. This empirical observation has been reported by others and is due to a collapse in which the learned fingerprints of all polymers, even chemically distinct ones, converge.^54,55^ However, as evidenced by the impressive performance of the MT polyGNN models on a vast majority of properties, the inability to learn block- or chain-level features is often ameliorated by the ability to learn lower-level features that go beyond those currently present in the handcrafted PG fingerprint. Still, the development of techniques that encourage GNNs to surpass the message passing threshold is a critical next step. We leave this task for future work.

## 4 Summary and Outlook

In summary, we have produced polyGNN—the first-ever protocol that integrates polymer feature learning from SMILES strings and other relevant features, invariant transformations, data augmentation, and multitask learning. Through careful comparison, we show that our protocol culminates in ultrafast polymer fingerprinting and accurate property prediction over the most comprehensive array of chemistries and properties studied to date. The gains in speed are essential when screening large candidate sets (e.g., millions or billions of polymers) and/or when computational resources are limited. Our approach is especially accurate when the training data set size is moderate to large. Even with data sets containing less than 300 points, our approach is at least competitive with presently adopted methods in a majority of cases.

Looking ahead, though polyGNNs perform remarkably well in the experiments tried here, handcrafted polymer fingerprints have advantages. In tasks where chain- or block-level features are essential, handcrafted fingerprinting approaches may yield the best model accuracy. Advances in the optimization of graph neural networks are needed to make the accuracy of polyGNNs competitive in these tasks. Finally, a handcrafted feature is, by definition, interpretable. In contrast, the features learned by the polyGNNs presented here are not interpretable. Following the work of others,^56^ future polyGNN architectures may incorporate attention mechanisms for partial interpretability. However, the interpretability of polyGNN features at the level of handcrafted features will require further innovation. Despite these shortcomings, we anticipate that the adoption of polyGNNs and related approaches will increase as they unlock the ability to screen truly massive polymer libraries at scale.

## 5 Public Use

The sources of data used in this work and the availability of each source is reported in the paper. The code used to train our polyGNN models is available at github.com/Ramprasad-Group/polygnn for academic use.

## Supporting Information Available

Data breakdown, polyGNN2 Encoder, polyGNN architecture, handcrafted PG models, training procedure, and extended results (PDF)

## References

[ref1] Baldwin A. F.; et al. Poly(dimethyltin glutarate) as a Prospective Material for High Dielectric Applications. Adv. Mater. 2015, 27, 346–351. 10.1002/adma.201404162. 25420940

[ref2] Mannodi-Kanakkithodi A.; Chandrasekaran A.; Kim C.; Huan T. D.; Pilania G.; Botu V.; Ramprasad R. Scoping the polymer genome: A roadmap for rational polymer dielectrics design and beyond. Mater. Today 2018, 21, 785–796. 10.1016/j.mattod.2017.11.021.

[ref3] Hu Y.; Zhao W.; Wang L.; Lin J.; Du L. Machine-Learning-Assisted Design of Highly Tough Thermosetting Polymers. ACS Appl. Mater. Interfaces 2022, 14, 55004 10.1021/acsami.2c14290. 36456181

[ref4] Doan Tran H.; Kim C.; Chen L.; Chandrasekaran A.; Batra R.; Venkatram S.; Kamal D.; Lightstone J. P.; Gurnani R.; Shetty P.; Ramprasad M.; Laws J.; Shelton M.; Ramprasad R. Machine-learning predictions of polymer properties with Polymer Genome. J. Appl. Phys. 2020, 128, 171104 10.1063/5.0023759.

[ref5] Kuenneth C.; Rajan A. C.; Tran H.; Chen L.; Kim C.; Ramprasad R. Polymer informatics with multi-task learning. Patterns 2021, 2, 100238 10.1016/j.patter.2021.100238. 33982028 PMC8085610

[ref6] Barnett J. W.; Bilchak C. R.; Wang Y.; Benicewicz B. C.; Murdock L. A.; Bereau T.; Kumar S. K. Designing exceptional gas-separation polymer membranes using machine learning. Science Advances 2020, 6, eaaz4301 10.1126/sciadv.aaz4301. 32440545 PMC7228755

[ref7] Patel R. A.; Borca C. H.; Webb M. A. Featurization strategies for polymer sequence or composition design by machine learning. Molecular Systems Design & Engineering 2022, 7, 661–676. 10.1039/D1ME00160D.

[ref8] Ma R.; Luo T. PI1M: A benchmark database for polymer informatics. J. Chem. Inf. Model. 2020, 60, 4684–4690. 10.1021/acs.jcim.0c00726. 32986418

[ref9] Ruddigkeit L.; Van Deursen R.; Blum L. C.; Reymond J. L. Enumeration of 166 billion organic small molecules in the chemical universe database GDB-17. J. Chem. Inf. Model. 2012, 52, 2864–2875. 10.1021/ci300415d. 23088335

[ref10] Franceschetti A.; Zunger A. The inverse band-structure problem of finding an atomic configuration with given electronic properties. Nature 1999 402:6757 1999, 402, 60–63. 10.1038/46995.

[ref11] Batra R.; Song L.; Ramprasad R. Emerging materials intelligence ecosystems propelled by machine learning. Nature Reviews Materials 2021, 6, 655 10.1038/s41578-020-00255-y.

[ref12] Gurnani R.; Kamal D.; Tran H.; Sahu H.; Scharm K.; Ashraf U.; Ramprasad R. polyG2G: A Novel Machine Learning Algorithm Applied to the Generative Design of Polymer Dielectrics. Chem. Mater. 2021, 33, 7008–7016. 10.1021/acs.chemmater.1c02061.

[ref13] Batra R.; Dai H.; Huan T. D.; Chen L.; Kim C.; Gutekunst W. R.; Song L.; Ramprasad R. Polymers for Extreme Conditions Designed Using Syntax-Directed Variational Autoencoders. Chem. Mater. 2020, 32, 10489–10500. 10.1021/acs.chemmater.0c03332.

[ref14] Kim C.; Batra R.; Chen L.; Tran H.; Ramprasad R. Polymer design using genetic algorithm and machine learning. Comput. Mater. Sci. 2021, 186, 110067 10.1016/j.commatsci.2020.110067.

[ref15] Yao Z.; Sánchez-Lengeling B.; Bobbitt N. S.; Bucior B. J.; Kumar S. G. H.; Collins S. P.; Burns T.; Woo T. K.; Farha O. K.; Snurr R. Q.; Aspuru-Guzik A. Inverse design of nanoporous crystalline reticular materials with deep generative models. Nature Machine Intelligence 2021, 3, 76–86. 10.1038/s42256-020-00271-1.

[ref16] Zunger A. Inverse design in search of materials with target functionalities. Nature Reviews Chemistry 2018, 2, 0121 10.1038/s41570-018-0121.

[ref17] Weininger D. SMILES, a chemical language and information system. 1. Introduction to methodology and encoding rules. J. Chem. Inf. Comput. Sci. 1988, 28, 31–36. 10.1021/ci00057a005.

[ref18] Lin T. S.; Coley C. W.; Mochigase H.; Beech H. K.; Wang W.; Wang Z.; Woods E.; Craig S. L.; Johnson J. A.; Kalow J. A.; Jensen K. F.; Olsen B. D. BigSMILES: AStructurally-Based Line Notation for Describing Macromolecules. ACS Central Science 2019, 5, 1523–1531. 10.1021/acscentsci.9b00476. 31572779 PMC6764162

[ref19] Chen G.; Tao L.; Li Y. Predicting polymers glass transition temperature by a chemical language processing model. Polymers 2021, 13, 1898 10.3390/polym13111898. 34200505 PMC8201381

[ref20] Gilmer J.; Schoenholz S. S.; Riley P. F.; Vinyals O.; Dahl G. E. Neural Message Passing for Quantum Chemistry. 34th International Conference on Machine Learning, ICML 2017 2017, 3, 2053–2070.

[ref21] Schütt K. T.; Kindermans P. J.; Sauceda H. E.; Chmiela S.; Tkatchenko A.; Müller K. R. SchNet: A continuous-filter convolutional neural network for modeling quantum interactions. Advances in Neural Information Processing Systems 2017, 992–1002.

[ref22] Jørgensen P. B.; Jacobsen K. W.; Schmidt M. N. Neural Message Passing with Edge Updates for Predicting Properties of Molecules and Materials. arXiv:1806.03146, 2018.

[ref23] Hy T. S.; Trivedi S.; Pan H.; Anderson B. M.; Kondor R. Predicting molecular properties with covariant compositional networks. J. Chem. Phys. 2018, 148, 241745 10.1063/1.5024797. 29960355

[ref24] Zhang S.; Liu Y.; Xie L. Molecular Mechanics-Driven Graph Neural Network with Multiplex Graph for Molecular Structures. arXiv:2011.07457, 2020.

[ref25] Ramakrishnan R.; Dral P. O.; Rupp M.; Von Lilienfeld O. A. Quantum chemistry structures and properties of 134 kilo molecules. Scientific Data 2014, 1, 140022 10.1038/sdata.2014.22. 25977779 PMC4322582

[ref26] Lecun Y.; Bengio Y.; Hinton G. Deep learning. Nature 2015 521:7553 2015, 521, 436–444. 10.1038/nature14539. 26017442

[ref27] Vaswani A.; Shazeer N.; Parmar N.; Uszkoreit J.; Jones L.; Gomez A. N.; Kaiser Å; Polosukhin I. Attention Is All You Need. Advances in Neural Information Processing Systems 2017, 5999–6009.

[ref28] Caruana R.; Pratt L.; Thrun S. Multitask Learning. Machine Learning 1997 28:1 1997, 28, 41–75. 10.1023/A:1007379606734.

[ref29] Brenner J. S. Sports specialization and intensive training in young athletes. Pediatrics 2016, 138, e20162148 10.1542/peds.2016-2148. 27573090

[ref30] Jørgensen P. B.; Mesta M.; Shil S.; García Lastra J. M.; Jacobsen K. W.; Thygesen K. S.; Schmidt M. N. Machine learning-based screening of complex molecules for polymer solar cells. J. Chem. Phys. 2018, 148.241735 10.1063/1.5023563 29960358

[ref31] Zeng M.; Kumar J. N.; Zeng Z.; Savitha R.; Chandrasekhar V. R.; Hippalgaonkar K. Graph Convolutional Neural Networks for Polymers Property Prediction. arXiv:1811.06231, 2018.

[ref32] St. John P. C.; Phillips C.; Kemper T. W.; Wilson A. N.; Guan Y.; Crowley M. F.; Nimlos M. R.; Larsen R. E. Message-passing neural networks for high-throughput polymer screening. J. Chem. Phys. 2019, 150, 234111 10.1063/1.5099132. 31228909

[ref33] Hatakeyama-Sato K.; Tezuka T.; Umeki M.; Oyaizu K. AI-Assisted Exploration of Superionic Glass-Type Li+ Conductors with Aromatic Structures. J. Am. Chem. Soc. 2020, 142, 3301–3305. 10.1021/jacs.9b11442. 31939282

[ref34] Mohapatra S.; An J.; Gómez-Bombarelli R. Chemistry-informed macromolecule graph representation for similarity computation, unsupervised and supervised learning. Machine Learning: Science and Technology 2022, 3, 015028 10.1088/2632-2153/ac545e.

[ref35] Aldeghi M.; Coley C. W. A graph representation of molecular ensembles for polymer property prediction. Chemical Science 2022, 13, 10486–10498. 10.1039/D2SC02839E. 36277616 PMC9473492

[ref36] Antoniuk E. R.; Li P.; Kailkhura B.; Hiszpanski A. M. Representing Polymers as Periodic Graphs with Learned Descriptors for Accurate Polymer Property Predictions. J. Chem. Inf. Model. 2022, 62, 5435 10.1021/acs.jcim.2c00875. 36315033

[ref37] Gurnani R.; Yu Z.; Kim C.; Sholl D. S.; Ramprasad R. Interpretable Machine Learning-Based Predictions of Methane Uptake Isotherms in MetalOrganic Frameworks. Chem. Mater. 2021, 33, 3543–3552. 10.1021/acs.chemmater.0c04729.

[ref38] Huan T. D.; Mannodi-Kanakkithodi A.; Ramprasad R. Accelerated materials property predictions and design using motif-based fingerprints. Physical Review B - Condensed Matter and Materials Physics 2015, 92, 014106 10.1103/PhysRevB.92.014106.

[ref39] Huan T. D.; Mannodi-Kanakkithodi A.; Kim C.; Sharma V.; Pilania G.; Ramprasad R. A polymer dataset for accelerated property prediction and design. Scientific Data 2016 3:1 2016, 3, 160012 10.1038/sdata.2016.12. PMC4772654 26927478

[ref40] Sharma V.; Wang C.; Lorenzini R. G.; Ma R.; Zhu Q.; Sinkovits D. W.; Pilania G.; Oganov A. R.; Kumar S.; Sotzing G. A.; Boggs S. A.; Ramprasad R. Rational design of all organic polymer dielectrics. Nature Communications 2014 5:1 2014, 5, 4845 10.1038/ncomms5845. 25229753

[ref41] Park J. Y.; Paul D. R. Correlation and prediction of gas permeability in glassy polymer membrane materials via a modified free volume based group contribution method. J. Membr. Sci. 1997, 125, 23–39. 10.1016/S0376-7388(96)00061-0.

[ref42] Kim C.; Chandrasekaran A.; Huan T. D.; Das D.; Ramprasad R. Polymer Genome: A Data-Powered Polymer Informatics Platform for Property Predictions. J. Phys. Chem. C 2018, 122, 17575–17585. 10.1021/acs.jpcc.8b02913.

[ref43] Zhu G.; Kim C.; Chandrasekarn A.; Everett J. D.; Ramprasad R.; Lively R. P. Polymer genome-based prediction of gas permeabilities in polymers. Journal of Polymer Engineering 2020, 40, 451–457. 10.1515/polyeng-2019-0329.

[ref44] Chen L.; Kim C.; Batra R.; Lightstone J. P.; Wu C.; Li Z.; Deshmukh A. A.; Wang Y.; Tran H. D.; Vashishta P.; Sotzing G. A.; Cao Y.; Ramprasad R. Frequency-dependent dielectric constant prediction of polymers using machine learning. npj Computational Materials 2020 6:1 2020, 6, 61 10.1038/s41524-020-0333-6.

[ref45] Lightstone J. P.; Chen L.; Kim C.; Batra R.; Ramprasad R. Refractive index prediction models for polymers using machine learning. J. Appl. Phys. 2020, 127, 215105 10.1063/5.0008026.

[ref46] Venkatram S.; Batra R.; Chen L.; Kim C.; Shelton M.; Ramprasad R. Predicting Crystallization Tendency of Polymers Using Multifidelity Information Fusion and Machine Learning. J. Phys. Chem. B 2020, 124, 6046–6054. 10.1021/acs.jpcb.0c01865. 32539396

[ref47] Brandrup J.; Immergut E. H.; Grulke E. A. Polymer Handbook, 4th ed.; John Wiley & Sons: New York, 1999.

[ref48] Barton A. F. M. CRC Handbook of Solubility Parameters and Other Cohesion Parameters, 2nd ed.; Routledge, 2013; p 768.

[ref49] Bicerano J. Prediction of Polymer Properties; Marcel Dekker, Inc.: New York, 2002.

[ref50] Otsuka S.; Kuwajima I.; Hosoya J.; Xu Y.; Yamazaki M. PoLyInfo: Polymer Database for Polymeric Materials Design. 2011 International Conference on Emerging Intelligent Data and Web Technologies (EIDWT); Tirana, 2011; pp 22–29.

[ref51] Crow Polymer Properties Database. https://polymerdatabase.com/, accessed March 13, 2022.

[ref52] RDKit, Open Source Toolkit for Cheminformatics. https://www.rdkit.org/, accessed March 13, 2022.

[ref53] He K.; Zhang X.; Ren S.; Sun J. Deep residual learning for image recognition. Pattern Recognition 2016, 770–778.

[ref54] Godwin J.; Schaarschmidt M.; Gaunt A.; Sanchez-Gonzalez A.; Rubanova Y.; Veličković P.; Kirkpatrick J.; Battaglia P. Simple GNN Regularisation for 3D Molecular Property Prediction & Beyond. arXiv:2106.07971, 2021.

[ref55] Chen D.; Lin Y.; Li W.; Li P.; Zhou J.; Sun X. Measuring and Relieving the Over-smoothing Problem for Graph Neural Networks from the Topological View. AAAI 2020 - 34th AAAI Conference on Artificial Intelligence 2020, 34, 3438–3445. 10.1609/aaai.v34i04.5747.

[ref56] Veličković P.; Casanova A.; Liò P.; Cucurull G.; Romero A.; Bengio Y. Graph Attention Networks. 6th International Conference on Learning Representations, ICLR 2018 - Conference Track Proceedings; 2017.

