# Supplementary material for: Polymer Informatics at Scale with Multitask Graph Neural Networks
Source: Chem Mater. 2023 Feb 15;35(4):1560–7. doi: 10.1021/acs.chemmater.2c02991 (PMC9979603; doi:10.1021/acs.chemmater.2c02991)
Supplement: Supplementary file 1 — cm2c02991_si_001.pdf [file cm2c02991_si_001.pdf]

Supporting Information

Polymer informatics at-scale with multitask graph neural networks

Rishi Gurnani, Christopher Kuenneth, Aubrey Toland, Rampi Ramprasad

School of Materials Science and Engineering, Georgia Institute of Technology, 30332
Atlanta, Georgia, United States

S1 Data breakdown

Our data set includes the permeability μ of six gases $g \in \{\text{He}, \text{H}_2, \text{CO}_2, \text{O}_2, \text{N}_2, \text{CH}_4\}$. The number of data points per gas is: 281 for He, 288 for H_2, 342 for CO_2, 380 for CH_4, 431 for N_2, and 436 for O_2. Our experimental dielectric constant ϵ_{f} data contains measurements at nine frequencies $f \in \{1.78, 2, 3, 4, 5, 6, 7, 9, 15\}$ in $\log_{10}$Hz. The number of data points per frequency is: 51 for $10^{1.78}$ Hz, 77 for 10^2 Hz, 172 for 10^3 Hz, 124 for 10^4 Hz, 66 for 10^5 Hz, 158 for 10^6 Hz, 20 for 10^7 Hz, 12 for 10^9 Hz, 507 for 10^{15} Hz.

S2 polyGNN2 Encoder

The input to the polyGNN2 Encoder is a repeat unit. From this repeating unit, the trimer graph (shown in Figure S1) is created. Then, the trimer graph is featurized. The atoms, bonds, and features corresponding to the central repeat unit of the trimer graph are used to create the periodic graph. Thus, the initial fingerprint of the periodic graph is always invariant to addition and subtraction, as shown for the example of polyacetylene in Figure S1. However, the polyGNN2 Encoder is slower than the polyGNN Encoder. While the polyGNN Encoder takes 26 seconds to featurize the graphs of 13 338 polymers, the polyGNN2 Encoder takes 37 seconds to featurize this set of polymer graphs.

S3 polyGNN architecture

polyGNNs contain three modules: the Encoder, Message Passing Block, and the Estimator. The inputs to polyGNN are a polymer repeat unit and a property of interest (represented by the property's associated selector vector). The two outputs of a polyGNN model are the repeat unit's fingerprint and the value of the property of interest.

In the Encoder, the repeat unit is first converted to a periodic graph, with each atom as a node and each bond as an edge. Then, each node and edge in the graph are given an initial fingerprint. After the graph elements have been assigned their initial features, the graph is passed to the Message Passing Block. Here, "messages" between neighboring atoms are iteratively passed along chemical bonds. After each iteration, every node fingerprint is updated using the messages, while each bond fingerprint remains the same. The message passed from atom j to atom i at time step k is calculated according to Eq. 1.

$$\boldsymbol{m}_{i,j}^{(k)} = \phi^{(k)}\left(\boldsymbol{x}_i^{(k)}, \boldsymbol{x}_j^{(k)}, \boldsymbol{e}_{i,j}\right) \tag{1}$$

where each $\phi^{(k)}$ is a parameterized function, $\boldsymbol{x}_i^{(k)}$ and $\boldsymbol{x}_j^{(k)}$ are the encodings of neighboring atoms after time step k, and $\boldsymbol{e}_{i,j}$ is the fingerprint of the bond that joins atoms i, j. $\boldsymbol{m}_{i,j}^{(k)} = 0$ if i, j do not share a chemical bond. After initialization, each node receives messages from all of its neighbors. These messages are aggregated by some permutation-invariant function $\mathcal{I}$ (e.g., `sum`, `mean`, `max`). We use the `sum` in this

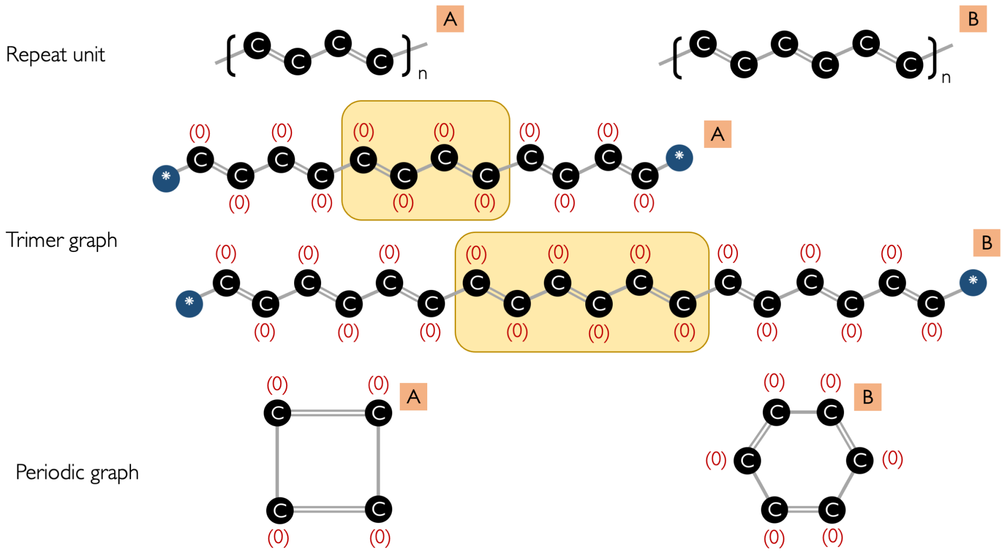

Figure S1: Two equivalent repeat units ("A" and "B", where "A" and "B" refer to four and six atom repeat units, respectively) of polyacetylene and their corresponding trimer and periodic graphs. Each repeat unit is converted to a trimer graph. Each node (i.e., heavy atom) in the trimer graph is featurized. Each atom is labeled with a zero if the atom is aliphatic or is labeled with a one if the atom is aromatic. Other atomic features and all bond features are not shown for visual clarity. The atoms, bonds, and features at the center of the trimer graph (shaded in yellow) are used to form the periodic graph.

work. The aggregated message, along with the current node encoding, is used to *update* the node encoding. The node update process is defined in Equation 2.

$$\boldsymbol{x}_i^{(k)} = \chi^{(k)}\left(\boldsymbol{x}_i^{(k-1)}, \mathcal{I}(\{\boldsymbol{m}_{i,j} \forall j \in [\![1, N_p]\!]\})\right) + x_i^{(k-2)} \tag{2}$$

where each $\chi^{(k)}$ is a parameterized function, p is a polymer, $[\![1, N_p]\!]$ is the set of integers between 1 and N_p, N_p is the number of atoms in the repeat unit of p, and $\boldsymbol{x}_i^{(k)} = 0, \forall k < 0$. Messages are passed for τ time steps, where τ is also the capacity in this work. The fingerprint of the entire polymer, $\boldsymbol{x}_p$, is calculated by the graph aggregation function $\mathcal{A}_g$, as shown in Eq. 3.

$$\boldsymbol{x}_p = \mathcal{A}_g(x_i^{(\tau)}, x_i^{(0)}) = \frac{1}{N_p}\sum_{i=1}^{N_p} \boldsymbol{x}_i^{(\tau)} + \boldsymbol{x}_i^{(0)} \tag{3}$$

Finally, $\boldsymbol{x}_p$ and the selector $\boldsymbol{s}$ can be passed to the Estimator. Here, these inputs are mapped to some polymer property prediction, y_p, via a parameterized function ψ. We implement ψ as a multilayer perceptron.

$$y_p = \psi(\boldsymbol{x}_p, \boldsymbol{s}) \tag{4}$$

During training, the parameters of all $\phi^{(k)}, \chi^{(k)}, \psi$ are learned *simultaneously*. As shown in Eq. 2, our update step leverages skip connections, which have been shown to improve the optimization of shallow layers in deep neural networks [1].

S4 Handcrafted PG models

The handcrafted PG models are made up of five MLP submodels trained using five-fold cross-validation (one submodel per fold). The input to each MLP is the handcrafted PG fingerprint of a given polymer repeat unit and a property selector. The output is the predicted property value.

S5 Training procedure

Each of the models discussed in the main text are ensemble models, composed of several submodels. The output of the ensemble is computed by a simple average of each submodel's output. For multitask ensembles, data for all properties within a group was combined, target values were scaled, and selectors were assigned as described in Section 2.1 of the main text. For single-task ensembles, properties were not combined into groups, MinMax scaling was not performed, and selectors were set to empty vectors. Next, to train and evaluate the model, the data was split according to the schematic in Figure S2 described below.

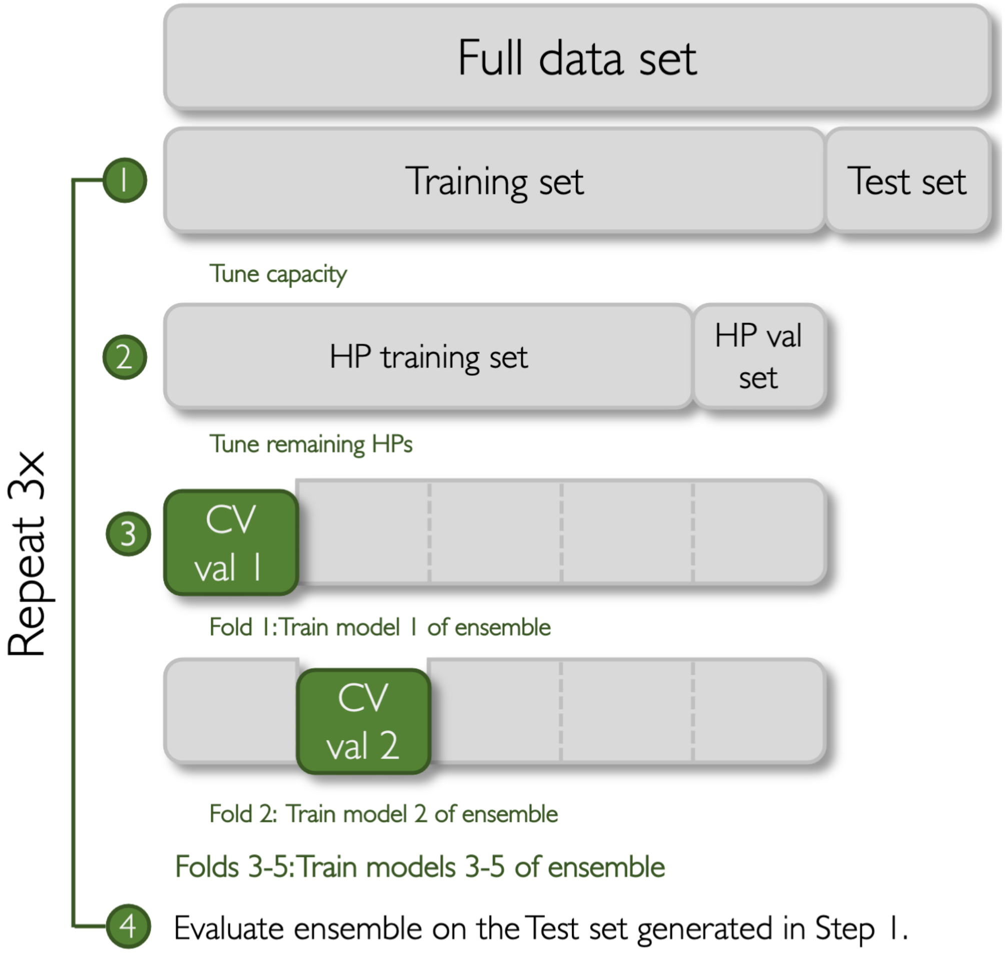

Figure S2: The various steps in our training and evaluation protocol. This protocol involved three runs, with three rounds of data splitting per run. All splits are random. "HP" stands for hyperparameter, "val" stands for validation, and "CV" stands for cross-validation.

First, the entire data set for each property was randomly cut into 80% training, 20% test splits three times. All subsequent steps were performed for each training-test set pair. Using the `NNDebugger` package [2], the optimal capacity was found by attempting to overfit the entire training data set. The data set was considered overfit if the R^2 value was greater than 0.97. If the data was not overfit, then the capacity corresponding to the highest R^2 value was used. The capacity range considered was between two and fourteen. The training data set was then divided into an 80% hyperparameter (HP) training set and a 20% HP validation set. The remaining HPs (batch size, learning rate, dropout percentage) were optimized using the package `scikit-optimize`. The set of HPs corresponding to the lowest RMSE on the HP validation set was considered optimal.

Finally, the training data set was split into five folds using cross-validation (CV), producing one CV train data set and one CV validation data set per fold. For each fold, the model's HPs were fixed as the optimal

HPs and the model's learnable parameters were fit to the CV train data set for 1000 epochs. At the end of 1000 epochs, the model parameters corresponding to the epoch with the lowest RMSE in the CV validation data set were chosen. After all five models were trained on their respective CV splits, the models were placed in an ensemble. The ensemble was used to make predictions of the test set, so far completely unseen by the ensemble during HP optimization or model training with CV.

All neural network architectures used dropout layers, fully connected layers, and Leaky ReLU activations (with a negative slope equal to 0.01). All architectures were created using `PyTorch` and/or `PyTorch Geometric`. The weights of all models were optimized using the Adam optimizer and the mean squared error loss function. All weights were initialized according to a Xavier uniform distribution [3] with a gain of one. All biases were initialized using the default `PyTorch` setting.

S6 Extended Results

We computed both the root-mean-squared-error (RMSE) and Pearson correlation coefficient (R^2) of each trained ensemble on unseen data for each property. The RMSE values are tabulated in the main body and the R^2 values are tabulated in Table S1.

As discussed in the main body, we desire that the final output of each polyGNN model is approximately invariant to addition and subtraction. In other words, the variance in predictions between a set of equivalent repeat units should be low. As shown in Table S2, we find that our proposed data set augmentation does lead to a significant decrease in prediction variance a majority of the time.

Table S2 contains the average variance of models trained with and without augmentation, $\widehat{var}_{\text{augment}}$ and $\widehat{var}_{\text{no augment}}$, respectively, on each of the 36 properties studied in this work. We define

$$\widehat{var} = \frac{1}{|\mathcal{P}|} \sum_{p \in \mathcal{P}} var(f(x), \forall x \in \mathcal{E}_p)$$

where $\mathcal{P}$ is a set of non-equivalent repeat units, var is the variance function, f is a machine learning model, and $\mathcal{E}_p$ is a set of repeat units related to p (a repeat unit in $\mathcal{P}$) by addition or subtraction. In this work, $\mathcal{P}$ is a set of 9 repeat units not seen by any model during training, and each $\mathcal{E}_p$ is composed of $p, 2p, 3p, 4p$ and $5p$. For example, if p is (−C−) then $\mathcal{E}_p$ = {(−C−), (−CC−), (−CCC−), (−CCCC−), (−CCCCC−)}.

Table S3 lists standard deviations of the data in our corpus, grouped by property.

References

[1] K. He, X. Zhang, S. Ren, and J. Sun, "Deep residual learning for image recognition," *Proceedings of the IEEE Computer Society Conference on Computer Vision and Pattern Recognition*, vol. 2016-December, pp. 770–778, 12 2016.

[2] R. Gurnani, "Debugging Neural Networks," 8 2021. [Online]. Available: https://nanohub.org/resources/netdebugger

[3] X. Glorot and Y. Bengio, "Understanding the difficulty of training deep feedforward neural networks," in *Proceedings of the Thirteenth International Conference on Artificial Intelligence and Statistics*, ser. Proceedings of Machine Learning Research, Y. W. Teh and M. Titterington, Eds., vol. 9. Chia Laguna Resort, Sardinia, Italy: PMLR, 2010, pp. 249–256. [Online]. Available: https://proceedings.mlr.press/v9/glorot10a.html

Property	MT polyGNN	MT handcrafted PG	ST polyGNN	ST handcrafted PG
λ*	0.106 ± 0.245	-0.177 ± 0.205	0.002 ± 0.209	-0.367 ± 0.678
T_{m}	0.840 ± 0.019	0.824 ± 0.023	0.757 ± 0.032	0.777 ± 0.018
T_{d}	0.746 ± 0.019	0.741 ± 0.021	0.662 ± 0.019	0.618 ± 0.059
T_{g}	0.913 ± 0.004	0.901 ± 0.004	0.885 ± 0.011	0.892 ± 0.004
E_{at}*	0.933 ± 0.057	0.614 ± 0.107	0.959 ± 0.011	0.879 ± 0.058
c_{p}*	0.729 ± 0.135	0.541 ± 0.280	0.734 ± 0.125	0.757 ± 0.142
O_{i}*	0.561 ± 0.092	0.475 ± 0.166	0.581 ± 0.057	0.596 ± 0.058
X_{e}*	0.227 ± 0.112	0.432 ± 0.137	0.143 ± 0.133	0.018 ± 0.210
V_{ff}*	0.380 ± 0.319	0.259 ± 0.355	0.534 ± 0.265	0.417 ± 0.351
X_{c}	0.519 ± 0.025	0.479 ± 0.078	0.397 ± 0.073	0.368 ± 0.072
ρ	0.894 ± 0.022	0.777 ± 0.034	0.900 ± 0.014	-4.126 ± 4.386
E_{a}*	0.738 ± 0.114	0.613 ± 0.105	0.781 ± 0.108	0.782 ± 0.079
E_{i}*	0.786 ± 0.156	0.659 ± 0.263	-6553.467 ± 11352.055	0.681 ± 0.162
E_{gb}*	0.928 ± 0.035	0.908 ± 0.040	0.839 ± 0.067	0.828 ± 0.046
E_{gc}	0.915 ± 0.002	0.896 ± 0.014	0.916 ± 0.005	0.895 ± 0.006
ϵ_0*	0.557 ± 0.358	0.652 ± 0.088	0.354 ± 0.404	0.683 ± 0.197
$\epsilon_{1.78}$*	0.842 ± 0.102	0.896 ± 0.088	-0.494 ± 0.851	0.224 ± 0.503
ϵ_2*	0.750 ± 0.252	0.807 ± 0.255	-12.141 ± 21.000	0.166 ± 0.302
ϵ_3*	0.819 ± 0.144	0.704 ± 0.199	0.203 ± 0.125	0.168 ± 0.116
ϵ_4*	0.861 ± 0.071	0.888 ± 0.062	0.346 ± 0.178	0.441 ± 0.232
ϵ_5*	0.797 ± 0.088	0.720 ± 0.149	-0.553 ± 2.096	-1.899 ± 3.879
ϵ_6*	0.519 ± 0.219	0.670 ± 0.078	-0.913 ± 1.112	0.013 ± 0.531
ϵ_{15}	0.860 ± 0.042	0.813 ± 0.060	0.815 ± 0.066	0.742 ± 0.084
n_{c}*	0.874 ± 0.061	0.737 ± 0.083	0.543 ± 0.307	0.532 ± 0.217
n_{e}	0.850 ± 0.020	0.826 ± 0.085	0.738 ± 0.083	0.437 ± 0.417
Y	0.501 ± 0.107	0.587 ± 0.094	0.428 ± 0.176	0.480 ± 0.073
σ_{ts}	0.638 ± 0.212	0.675 ± 0.155	0.499 ± 0.173	0.576 ± 0.152
δ_{s}*	0.770 ± 0.074	0.235 ± 0.180	0.536 ± 0.156	0.686 ± 0.045
μ_{He}*	0.969 ± 0.002	0.978 ± 0.007	0.877 ± 0.037	0.891 ± 0.023
$\mu_{\mathrm{H_2}}$*	0.983 ± 0.002	0.988 ± 0.002	0.914 ± 0.018	0.857 ± 0.041
$\mu_{\mathrm{CO_2}}$	0.980 ± 0.006	0.981 ± 0.006	0.866 ± 0.027	0.779 ± 0.174
$\mu_{\mathrm{CH_4}}$	0.986 ± 0.005	0.990 ± 0.004	0.897 ± 0.029	0.881 ± 0.009
$\mu_{\mathrm{N_2}}$	0.985 ± 0.003	0.988 ± 0.003	0.833 ± 0.070	0.844 ± 0.034
$\mu_{\mathrm{O_2}}$	0.981 ± 0.003	0.987 ± 0.002	0.845 ± 0.028	-6.798 ± 13.235

Table S1: Average R^2 plus/minus one standard deviation on unseen test data. Starred properties contain 300 or fewer data points.

Property	$\widehat{var}_{\text{no augment}}$	$\widehat{var}_{\text{augment}}$	$\frac{\widehat{var}_{\text{no augment}}}{\widehat{var}_{\text{augment}}}$
E_{a}	0.0237	0.0086	2.763
E_{at}	0.0055	0.0014	3.840
E_{i}	0.0144	0.0123	1.171
E_{gb}	0.0332	0.0177	1.874
E_{gc}	0.0414	0.0310	1.338
ϵ_0	0.0016	0.0021	0.7769
n_{c}	0.0009	0.0020	0.4282
c_{p}	0.0040	0.0062	0.6490
σ_{ts}	0.0046	0.0011	4.318
T_{g}	0.0088	0.0009	9.969
T_{m}	0.0088	0.0018	4.807
Y	0.0049	0.0009	5.313
X_{e}	0.0214	0.0145	1.480
X_{c}	0.0186	0.0083	2.225
$\epsilon_{1.78}$	0.0010	0.0010	0.9407
ϵ_{15}	0.0031	0.0018	1.765
ϵ_2	0.0014	0.0009	1.631
ϵ_3	0.0010	0.0005	2.156
ϵ_4	0.0010	0.0012	0.8613
ϵ_5	0.0019	0.0014	1.432
ϵ_6	0.0010	0.0026	0.3841
ϵ_7	0.0011	0.0012	0.8877
ϵ_9	0.0007	0.0011	0.6105
V_{ff}	0.0070	0.0079	0.8951
O_{i}	0.0029	0.0027	1.089
μ_{CH_4}	0.0167	0.0093	1.788
μ_{CO_2}	0.0155	0.0088	1.764
μ_{H_2}	0.0165	0.0092	1.795
μ_{He}	0.0162	0.0066	2.454
μ_{N_2}	0.0178	0.0113	1.581
μ_{O_2}	0.0190	0.0117	1.630
n_{e}	0.0036	0.0021	1.699
ρ	0.0037	0.0022	1.690
δ_{s}	0.0096	0.0073	1.330
λ	0.0079	0.0014	5.774
T_{d}	0.0123	0.0005	26.71

Table S2: The average variance of models trained with and without augmentation. The unit of each property is given in Table S3.

Property	σ
λ	0.0653 W/mK
T_{m}	109.3 K
T_{d}	114.7 K
T_{g}	109.0 K
E_{at}	0.470 eV/atom
c_{p}	0.374 J/gK
O_{i}	13.10 %
X_{e}	18.3 %
V_{ff}	0.0477
X_{c}	23.7 %
ρ	0.1991 g/cc
E_{a}	0.777 eV
E_{i}	1.101 eV
E_{gb}	1.760 eV
E_{gc}	1.561 eV
ϵ_0	0.726
$\epsilon_{1.78}$	1.388
ϵ_2	1.331
ϵ_3	1.276
ϵ_4	0.991
ϵ_5	1.039
ϵ_6	0.854
ϵ_{15}	0.359
n_{c}	0.1713
n_{e}	0.1142
Y	1.401 MPa
σ_{ts}	40.9 MPa
δ_{s}	2.64 $\sqrt{\mathrm{MPa}}$
μ_{He}	0.806 Barrer
$\mu_{\mathrm{H_2}}$	0.993 Barrer
$\mu_{\mathrm{CO_2}}$	1.207 Barrer
$\mu_{\mathrm{CH_4}}$	1.050 Barrer
$\mu_{\mathrm{N_2}}$	0.950 Barrer
$\mu_{\mathrm{O_2}}$	1.018 Barrer

Table S3: The standard deviation (σ) of data in our corpus, grouped by property.
